# Morphological and Metabolic Changes in the Nigro-Striatal Pathway of Synthetic Proteasome Inhibitor (PSI)-Treated Rats: A MRI and MRS Study

**DOI:** 10.1371/journal.pone.0056501

**Published:** 2013-02-19

**Authors:** Stefano Delli Pizzi, Cosmo Rossi, Vincenzo Di Matteo, Ennio Esposito, Simone Guarnieri, Maria Addolorata Mariggiò, Raffaella Franciotti, Massimo Caulo, Astrid Thomas, Marco Onofrj, Armando Tartaro, Laura Bonanni

**Affiliations:** 1 ITAB, “G. D’Annunzio University”, Chieti, Italy; 2 Aging Research Center, Ce.S.I., “Gabriele d’Annunzio” University Foundation, Chieti, Italy; 3 Laboratory of Neurophysiology, Istituto di Ricerche Farmacologiche Mario Negri, Consorzio Mario Negri Sud, Santa Maria Imbaro (Chieti), Italy; 4 Department of Neuroscience and Imaging and CE.S.I. Aging Research Center, University G.d’Annunzio of Chieti-Pescara, Italy; INSERM/CNRS, France

## Abstract

Systemic administration of a Synthetic Proteasome Inihibitor (PSI) in rats has been described as able to provide a model of Parkinson’s disease (PD), characterized by behavioral and biochemical modifications, including loss of dopaminergic neurons in the substantia nigra (SN), as assessed by post-mortem studies. With the present study we aimed to assess *in-vivo* by Magnetic Resonance (MR) possible morphological and metabolic changes in the nigro-striatal pathway of PSI-treated rats. 10 animals were subcutaneously injected with PSI 6.0 mg/kg dissolved in DMSO 100%. Injections were made thrice weekly over the course of two weeks. 5 more animals injected with DMSO 100% with the same protocol served as controls. The animals underwent MR sessions before and at four weeks after the end of treatment with either PSI or vehicle. MR Imaging was performed to measure SN volume and Proton MR Spectroscopy (^1^H-MRS) was performed to measure metabolites changes at the striatum. Animals were also assessed for motor function at baseline and at 4 and 6 weeks after treatment. Dopamine and dopamine metabolite levels were measured in the striata at 6 weeks after treatment. PSI-treated animals showed volumetric reduction of the SN (p<0.02) at 4 weeks after treatment as compared to baseline. Immunofluorescence analysis confirmed MRI changes in SN showing a reduction of tyrosine hydroxylase expression as compared to neuron-specific enolase expression. A reduction of N-acetyl-aspartate/total creatine ratio (p = 0.05) and an increase of glutamate-glutamine-γ amminobutirrate/total creatine were found at spectroscopy (p = 0.03). At 6 weeks after treatment, PSI-treated rats also showed motor dysfunction compared to baseline (p = 0.02), accompanied by dopamine level reduction in the striatum (p = 0.02). Treatment with PSI produced morphological and metabolic modifications of the nigro-striatal pathway, accompanied by motor dysfunction. MR demonstrated to be a powerful mean to assess *in-vivo* the nigro-striatal pathway morphology and metabolism in the PSI-based PD animal model.

## Introduction

In Parkinson’s Disease (PD), degeneration of the nigro-striatal dopaminergic pathway with cell loss in the substantia nigra (SN) and biochemical changes at the striatum are associated with intracellular accumulation of alpha-synuclein, at present considered the pathological hallmark of PD [1].

The mechanisms leading to accumulation of alpha synuclein are still largely unknown, but the appearance of alpha-synuclein inclusions has been associated to proteasome dysfunction [2]–[4].

According with this data, a rat model of PD, based on systemic injection of a synthetic proteasome inhibitor (PSI, Z-Ile-Glu(OtBU)-Ala-Leu-CHO) was recently proposed [2].

In the original description, the administration of PSI caused parkinsonism with progressive features of dopaminergic cell loss in the SN (as assessed by post-mortem studies) and decreased motor activity.

After this first description, many laboratories attempted to reproduce the model with controversial results [4]–[8]. The inconsistencies in observations related to the PSI-based animal model of PD have not been totally explained. Technical difficulties have been claimed as responsible for unsuccessful reproduction of the data, and the consequence has been the loss of interest for the model by some experienced laboratories [8]–[9].

Nevertheless, the concept of abnormal protein aggregation is still the focus of research on PD [10], and, even though cautious conclusions are demanded, we believe that PSI based models can unveil unexplored aspects of SN pathophysiology, as the publications of recent works, using PSI in combination with other compounds by different laboratories seem to confirm [11]–[12].

With the present study we aim to verify the ability of PSI to produce metabolic (dopamine level changes at the striatum) and morpho/metabolic modifications of the nigro-striatal pathway, akin to dysfunctions found in PD. To achieve our aim, we used Magnetic Resonance Imaging (MRI) and Proton Magnetic Resonance Spectroscopy (^1^H-MRS), which investigate *in vivo* the structural and metabolic modifications in the brain areas of interest and we compared imaging results to imnunocytochemical study of potential loss of nigral dopamine containing neurons.

Our second aim was consequentially to validate MR techniques as a tool able to analyze morphological changes and alterations in neuronal metabolite signatures in live animals related to neurodegeneration in a rat model of PD.

## Materials and Methods

Fifteen male Sprague-Dawley adult rats (250–290 g, 6 week old), were housed at the Ce.S.I., Animal facility, Chieti, Italy, under standard conditions and were provided with food and water ad libitum. All animal experiments were carried out with local ethical approval by Comitato Etico Interateneo per la Sperimentazione Animale (Inter-University Ethical Committee for animal experiments; 08/2010/CEISA/PROG/05) and care was taken to reduce any suffering.

### Proteasome Inhibitor Treatment

Ten animals were treated with the ubiquitin proteasome inhibitor (Z-lle-Glu(OtBu)-Ala-Leu-al; PSI) (Peptides International Inc, Kentucky,USA) [2].

Rats were subcutaneously (s.c.) injected with 6.0 mg/kg PSI [middle dosage between the dose reported in McNaught et al (3.0 mg/Kg PSI) [2] and the mean of reactive doses reported in Bukhatwa et al (10.0 mg/Kg PSI) [11] reconstituted with dimethyl sulfoxide (DMSO) 100% (freshly prepared solution of 810 µL DMSO in every 5 mg vial of PSI, for a volume of 200 µL per rat). Injections were made thrice weekly (Mon., Wed., Fri.) over the course of two weeks.

Five control animals were subcutaneously injected with DMSO 100% with the same time protocol applied for PSI-treated animals.

### Behavioral Assessment

All the animals were tested at baseline and at 4 and 6 weeks after treatments for presence, severity, and progression of motor dysfunction. Motor function was assessed by treadmill and tail suspension tests [13].

### MR Experiment

MR acquisitions were performed by adapting a horizontal bore 3T scanner (Philips Achieva, Philips Medical System, Best, the Netherlands) routinely employed for clinical use**,** with a dedicated animal coil (4-Channel High Resolution Animal Array, Ø 50 mm) provided by the manufacturer.

The animals underwent MR sessions before and at four weeks after the end of treatment with either PSI or vehicle. Before each MR session, rats were anesthetized with fenobarbital (50 mg/Kg).

At the end of each session a reference scout sequence was repeated to exclude possible head displacement during acquisition. A displacement of ≤10% of the maximum coronal brain diameter acquired (mean± SD 1.5±0.1 mm) along the three axes was considered as tolerable.

In each session, after scout and reference, T_2_-weighted turbo spin echo (T_2_-TSE) images were acquired in axial, coronal and sagittal rat planes to provide the anatomical rat brain images to place ^1^H-MRS voxels. High resolution T_2_-TSE images in coronal orientation were performed with matrix 64×120 pixels, FOV (ap,fh,rl) = 30×30×23 mm, slice thickness 2 mm, gap 0.1 mm, in-plane voxel size 0.2×0.2×2 mm, flip angle 90°, repetition time (TR) of 3150 ms, echo time (TE) of 80 ms. T_2_-TSE sagittal images were performed with matrix 120×93 pixels, FOV = 68×70×33 mm, slice thickness 3 mm, gap 0.2 mm, in-plane voxel size 0.45×0.45×3 mm, flip angle 90°, TR = 3000 ms, TE = 80 ms. T_2_-TSE axial images were acquired with T_2_-TSE sequence, matrix 120×90 pixels, FOV = 13×70×68 mm, slice thickness 2 mm, gap 1 mm, in-plane voxel size 0.15×0.15×2 mm, flip angle 90°, TR = 3000 ms, TE = 80 ms. T_2_*-weighted gradient echo (T_2_*-GE) images were also acquired in coronal orientation with the following scan parameters: matrix 168×167 pixels, FOV = 50×11×50 mm, slice thickness 1 mm, gap 0.1 mm, in-plane voxel size 0.3×0.3×1 mm, flip angle 18°, TR = 4500 ms, TE = 16 ms.

### MR Imaging

The SN, which represents the primary target of PD neuropathological cascade, was set as the main target region of the MRI study.

Since SN is characterized by local dishomogeneity due to ferromagnetic substances accumulation, especially in PD [14]–[16] T_2_*-GE weighted sequences were used due to their high sensitivity to substances characterized by elevated magnetic susceptibility [17]. Coronal T_2_*-GE images were acquired and evaluated to measure SN area. Cerebral cortex (CC) and whole brain (WB) areas were also evaluated to verify whether possible effects of treatments were limited to SN or spread to different brain areas, not directly involved in the pathological cascade of PD.

The three regions were identified on the basis of a brain atlas [18] and were manually [16] drawn with the Philips Extended MR Work Space 2.6.3.2. by two experienced readers unaware of which image they were analyzing (whether from pre or post-treatment condition).

The delimited area was subsequently automatically quantified by the Philips Extended MR Work Space 2.6.3.2.

**Figure 1:**
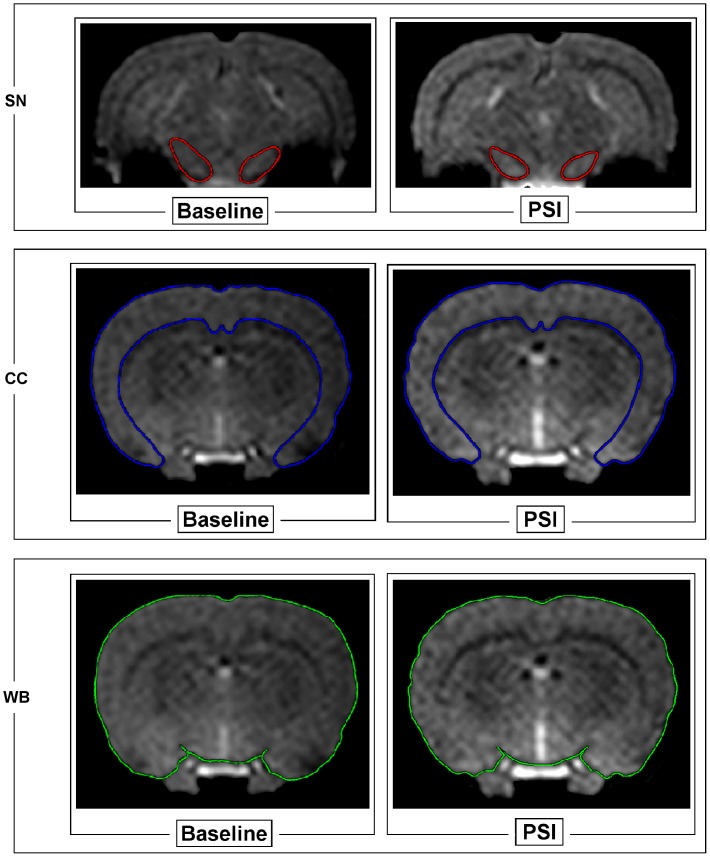
Coronal images of brain areas of a representative PSI-treated animal by using T_2_*-weighted gradient-echo sequences. Top panel shows substantia nigra area (SN, delimited by the red frame) before (left) and after (right) PSI treatment. Notice the rim of low T_2_* signal intensity which characterizes the external margin of SN. **Middle panel** shows cerebral cortex area (CC, delimited by the blue frame) before (left) and after (right) PSI treatment. **Bottom panel** shows whole brain area (WB, delimited by the green rim) before (left) and after (right) PSI treatment. CC and WB areas were drawn on a coronal slice passing through the nucleus striatum.

The external margin of SN is easily identifiable because of its intrinsic properties of low T_2_* signal intensity (**Figure 1** **, panel A**). CC and WB areas were measured on a coronal slice passing through the nucleus striatum (**Figure 1** **, panels B and C**). Particularly, the ventral CC boundaries were identified by using as reference the relative T_2_* signal hyperintensity in CC respect to white matter of callosum body and external capsule.

For each animal the areas of interest (SN, CC and WB) were measured in mm^2^ at baseline and after treatment in the two hemispheres and averaged. To correct for possible modifications of the whole brain (WB) area over the six weeks study, the values were expressed as SN/WB and CC/WB.

### Proton MR Spectroscopy

#### The nucleus striatum was the focus of the Proton MR spectroscopy study

**Figure 2:**
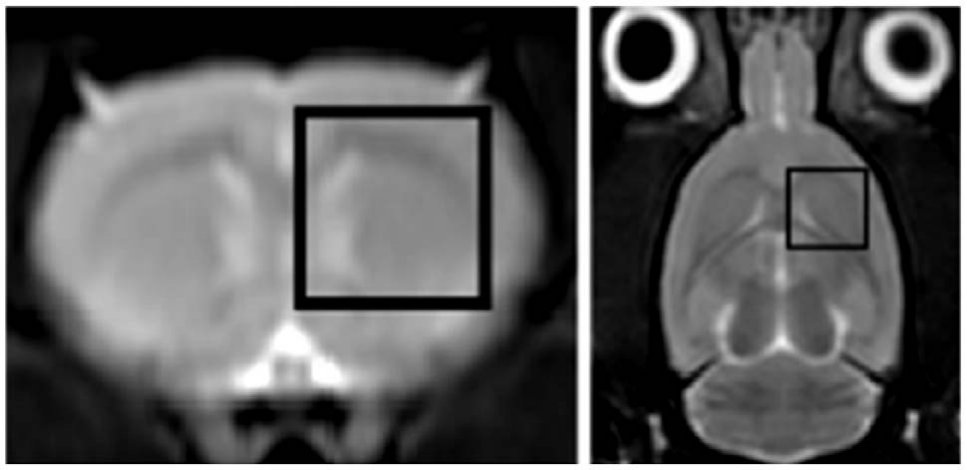
^1^H-MRS voxel on the nucleus striatum. Coronal and axial T_2_-weighted Turbo Spin Echo (T_2_-TSE) images of the rat brain showing a 5×5×5 mm^3^ ^1^H-Magnetic Resonance Spectroscopy (^1^H-MRS) voxel (delimited by the white perimeter) centered on the nucleus striatum.

^1^H-MRS 5×5×5 mm^3^ voxel was positioned on T_2_-TSE images and centered on the nucleus striatum (**Figure 2** **)**, in agreement with the rat brain atlas [18] and as widely reported in literature [19]–[20].

Point-resolved spectroscopy (PRESS) sequences (TR = 2000 ms, TE = 144 ms, 16-step phase-cycle and an averages for 256 scan) were performed with water suppression using chemically shift selective (CHESS) pulses. 1024 points were acquired with a spectral width of 2000 Hz.^ 1^H-MRS data analysis were performed by jMRUI version 4.0 [19]. Water suppressed spectra were filtered for removal of residual water by using the Hankel Lanczos Singular Values Decomposition (HLSVD) method [20]. Autophasing and baseline correction were performed. Frequency shifts were corrected using the NAA signal as a reference and a priori knowledge database (NAA, 2.02 ppm; Glx, 2.10–2.45 ppm; tCr; 3.03 ppm; tCho, 3.22 ppm) was constructed to put constraints on the Advanced Magnetic Resonance (AMARES) fitting algorithm [21] within jMRUI package. Peak shifts were restricted to ±5 ppm of the theoretical location. From each unsuppressed spectra, the area of the water peak was calculated by the same protocol to establish a reference signal to use as an internal standard [22]–[23]. All non-water signals were removed from the unsuppressed free-induction decays by using the HLSVD method.

### Brain Tissue Processing

Two weeks after the last MRI sessions (week 6 after treatment), right after the last behavioural test, all the animals were sacrificed by cervical dislocation. Brains were removed and split in two hemispheres. For each animal, one hemisphere (randomly selected) was cryoprotected by serial passages in sucrose in PBS, pH 7.4; first in 10% sucrose for 24 h and then in 30% sucrose for 2–5 days, then frozen in isopentane –45°C and then stored at –80°C for subsequent immunocytochemistry study. The contralateral hemisphere was immediately placed into ice-cold saline for subsequent HPLC analysis.

### Immunofluorescence Analysis

Possible degeneration of dopaminergic neurons in the SN following PSI treatment was evaluated by immunofluorescence analysis. Coronal sections (30 µm thickness) were cut using a cryostat microtome, mounted in gelatine-coated slices. For immunofluorescence, sections were washed with PBS and permeabilized with 0.5% Triton X-100 in PBS at room temperature for 10 min and incubated in 10% goat serum at room temperature for 1 hour followed by an overnight immunostaining at 4°C with a solution containing rabbit anti-tyrosine hydroxilase (TH) polyclonal antibody (dil. 1∶500, Abcam Limited Cambridge, UK) and chicken anti-neuronal specific enolase (NSE) (dil. 1∶1000, Millipore, Temecula, USA). The samples were washed thoroughly, incubated for 2 hour at 37°C with goat Alexa568-conjugated anti-rabbit IgG (dil. 1∶200, Molecular Probes) and Alexa488-conjugated anti-chiken IgY (dil. 1∶200, Sigma-Aldrich). The slides were dried, mounted and observed. Images were collected using a Zeiss LSM510 META confocal system (Carl Zeiss, Jena, Germany) connected to an inverted microscope (Zeiss Axiovert 200) equipped with 40X/1.4 PLAN NEOFLUAR oil immersion objective. For red fluorescence emission of Alexa-568-conjugated antibody, excitation was fixed at 543 nm and emission at 605–630 nm. For green fluorescence emission of the Alexa488-conjugated anti-chiken antibody, excitation was fixed at 488 nm and emission at 515–530 nm using a bandpass filter. Red and green channels were sequentially acquired (on track mode), to avoid signal overlapping. The laser potency, photo-multiply and pin-hole size were kept constant for all experiments. For each sample, at least 5 randomized fields were acquired in the SN using LSM software (Carl Zeiss) and off-line analyzed. For each image, the area deriving from red (TH) or green (NSE) fluorescence signal was measured using Zen 2011 software (Carl Zeiss).

### Measurements of Rat Striatal Dopamine and Dopamine Metabolites

The effects of PSI treatment on the amount of dopamine (DA) and the dopamine metabolite 3, 4-dihydroxyphenylacetic acid (DOPAC) in the striata, were evaluated by High-Performance Liquid Chromatography (HPLC) analysis.

For HPLC analysis, tissue samples were weighed, transferred into 1 mL antioxidant solution (0.1 N HClO_4_, 0.1% Na_2_S_2_O_5_, 0.01% Na_2_EDTA) containing internal standard (10 µl dihydroxybenzylamine 3 µM) and afterwards homogenized for 1 min by ultrasounds (vibra cell™ VC 50, Sonics & Materials Inc. Danbury, CT, USA) and then centrifuged (4224 ALC centrifuge, Milano, Italy) for 15 min at 12000 rotations/min and 4°C. The centrifuged was filtered through a membrane filter with a pore size of 0.45 µm (type Millex®-HV, 0.45 µm Syringe filters, Japan) before HPLC assay.

Dialysate samples were analyzed by reversed-phase HPLC coupled with electrochemical detection. The mobile phase was composed of 24 mM citric acid, 16 mM Na_2_HPO_4_, 0.19 mM Na_2_EDTA, 1.22 mM 1-eptansulfonic acid, and 17.5% methanol, adjusted to pH 2.8 with orthophosphoric acid. This mobile phase was delivered at 1 mL/min flow rate (LC-10 AD*vp* pump, Shimadzu Italia, Milano) through a Supelcosil™ column (LC-C8, 4.0×250 mm, 5 µm, Supelco, Bellefonte, PA, USA). Samples were injected manually into the HPLC and detection of DA and DOPAC was carried out with a coulometric detector (Coulochem II, ESA, Bedford, MA, USA) coupled to a dual electrode analytic cell (model 5014, Coulochem II, ESA, Bedford, MA, USA). The potential of the first electrode was set at 0 mV and the second at +400 mV. Under these conditions, the sensitivity for DA was 0.35 pg/20 µl with a signal to noise ratio of 3∶1.DA and DOPAC content in each sample was expressed as ng/g tissue. Data correspond to mean ± SEM values of absolute DA and DOPAC levels obtained in each experimental group.

### Statistical Analysis

For behavioural assessments and for measurements of rat striatal dopamine metabolites data were analyzed by analysis of variance (ANOVA), followed by the Fisher’s protected least significance difference *post hoc* test (Fisher’s PLSD) to allow multiple comparisons between groups.

For MRI and H^1^-MRS, data were analyzed by non-parametric Kruskal-Wallis test, followed by Wilcoxon and Mann-Whitney *post hoc* test to allow multiple comparisons within and between groups.

Student’s t-test was applied to analyze immunofluorescence data.

Intra- and inter-rater reliability tests were performed by non-parametric Kruskal-Wallis test, followed respectively by Wilcoxon and Mann-Whitney *post hoc* test to allow multiple comparisons within and between groups.

Statistical significance was set at p<0.05 for all the analyses performed.

All statistical analyses were performed with StatView™ version 5.0.1 (SAS Institute Inc., Cary, NC, USA).

## Results

### MR Experiments

All the animals were vital before and after all MR sessions.

In none of the animals a head displacement >10% of the maximum coronal brain diameter along the three axes was detected. Mean head displacement in the 15 studied animals was of 0.1±0.2 mm.

### MR Imaging

MR imaging estimated morphometric modifications of SN (**Figure 1** **, top panels)** and CC (**Figure 1** **, middle panels)** areas of PSI treated rats as compared to baseline (pre-treatment conditions).

Intra- and inter- rater reliability test showed no differences in the evaluation of SN, CC or WB areas (**Supplementary** **figure 1**).

**Figure 3:**
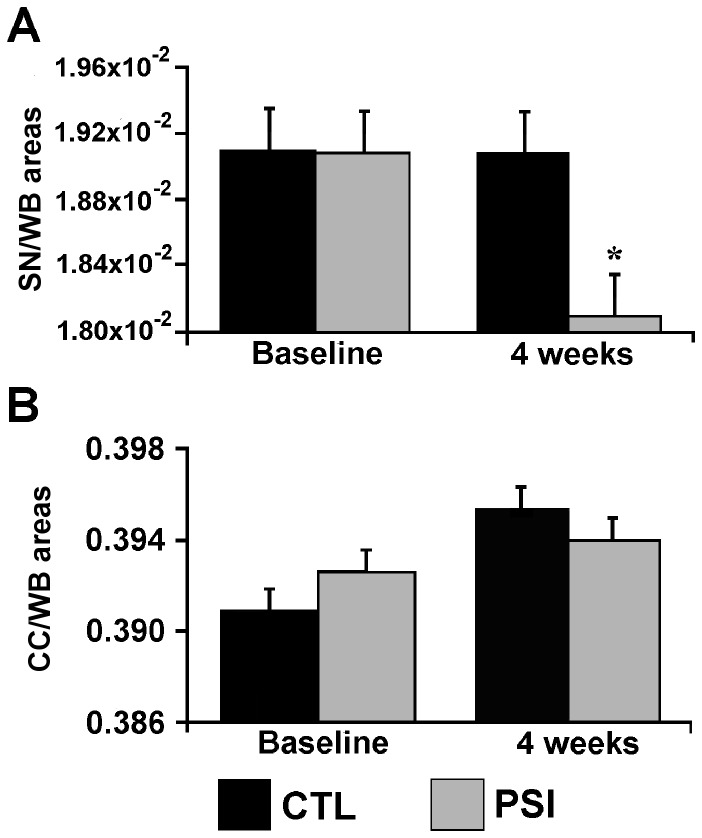
SN/WB and CC/WB areas modifications after PSI treatments. Bar graphs represent the size changes of the brain areas of interest. **Panel A** shows group mean ± standard error of the values of SN/WB areas before and after PSI (grey bars, n = 10) and DMSO (black bars, n = 5) treatments. **Panel B** shows group mean ± standard error of the values of CC/WB areas before and after PSI (grey bars, n = 10) and DMSO (black bars, n = 5) treatments. The significance level was set at p<0.05 and marked with a star.

At four weeks after PSI-treatment, a 6% reduction of the SN/WB area was evidenced, as compared with baseline condition (p = 0.02) (**Figure 3** **, panel A)**.

No change was found in CC/WB **(** **Figure 3** **, panel B)**.

No morphometric change was found either in the SN/WB or in the CC/WB of vehicle-treated animals at 4 weeks after treatment as compared to baseline (pre-treatment condition) (**Figures 3** **, panel A and B**).

### Proton MR Spectroscopy

**Figure 4:**
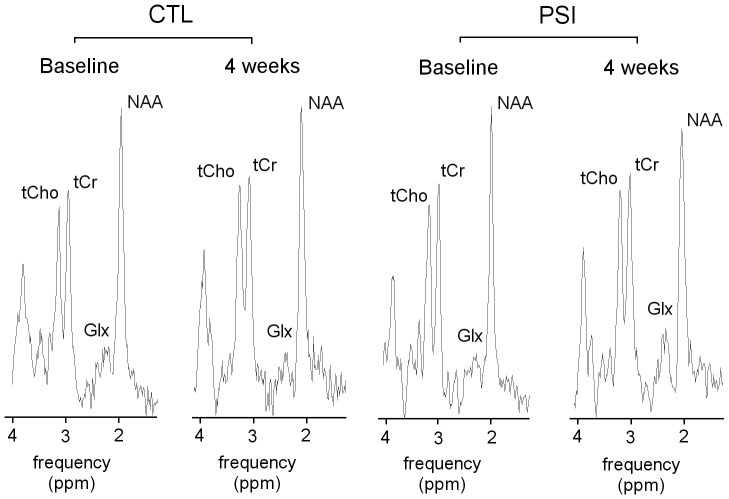
Representative ^1^H-MRS spectra acquired before and after treatments. Point-resolved spectroscopy (PRESS) sequences with CHESS water suppression were performed at an echo time (TE) of 144 ms to detect the contributions of N-acetyl aspartate (NAA), total creatine (tCr) total choline (tCho), Glx (which describes glutamine (Gln) and glutamate (Glu) contributions). The acquisition duration for each spectra was 12 min. Left panel shows results in a representative vehicle-treated animal at baseline and at 4 weeks after treatment. Right panel shows results in a representative PSI-treated animal at baseline and at 4 weeks after treatment.

Morphometric degeneration of SN after PSI treatment was accompanied by metabolites/tCr changes at the striatum (**Figures 4** **and** **5**). NAA/tCr was significantly reduced (p = 0.05); Glx/tCr was increased (p = 0,03). tCho/tCr resulted unchanged. Control animals visualized over a similar time frame demonstrated no changes in the levels of each metabolite/tCr at the striatum (**Figures 4** **and** **5** **)**.

**Figure 5:**
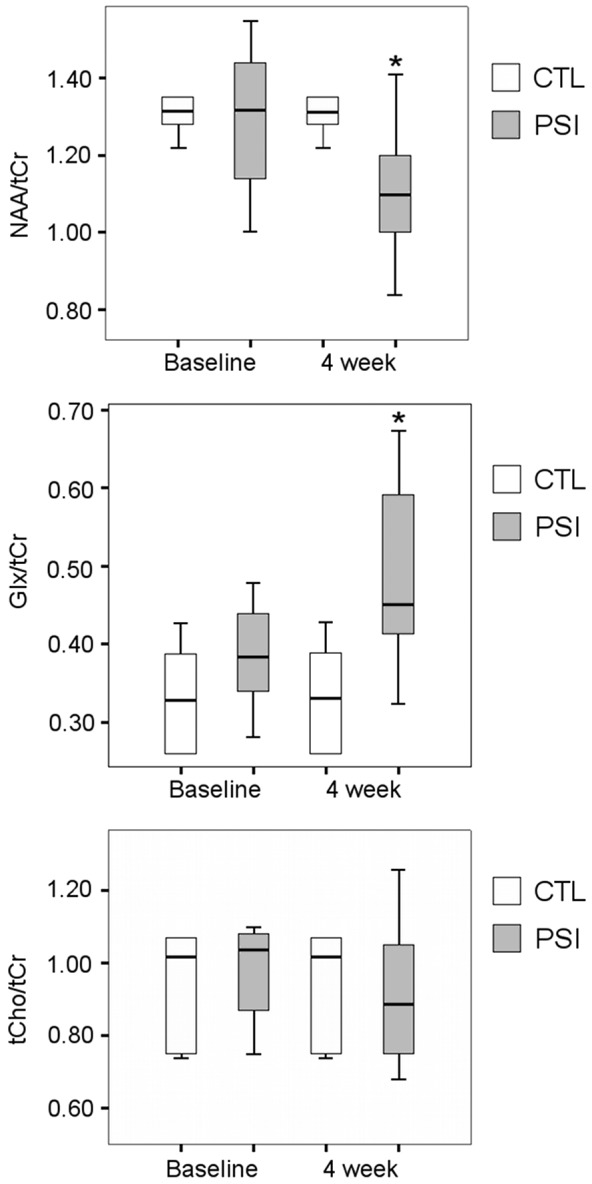
^1^H-MRS metabolite levels modifications in the nucleus striatum of treated animals. Box and Whiskers plot describes the distribution of the metabolites of interest quantified with in vivo ^1^H-MRS in the nucleus striatum of the studied animals and expressed as metabolite/tCr. Results from control animals are represented as white box (CTL, n = 5), results from PSI-treated animals are represented as grey box (PSI, n = 10). The bottom and top of the box show respectively the lower and upper quartiles; the bold band is the median; the ends of the whiskers show the minimum and the maximum value. Significant difference (p<0.05) is marked with a star. NAA = N-acetyl aspartate, tCr = total creatine, tCho = total choline, Glx = Gln (glutamine) and Glu (glutamate) contributions.

tCr levels were comparable in the treatment groups at baseline and after treatment and appeared to be stable in both groups of treatment during the study (**Supplementary** **Figure 2**).

### Behavioral Experiment

**Figure 6:**
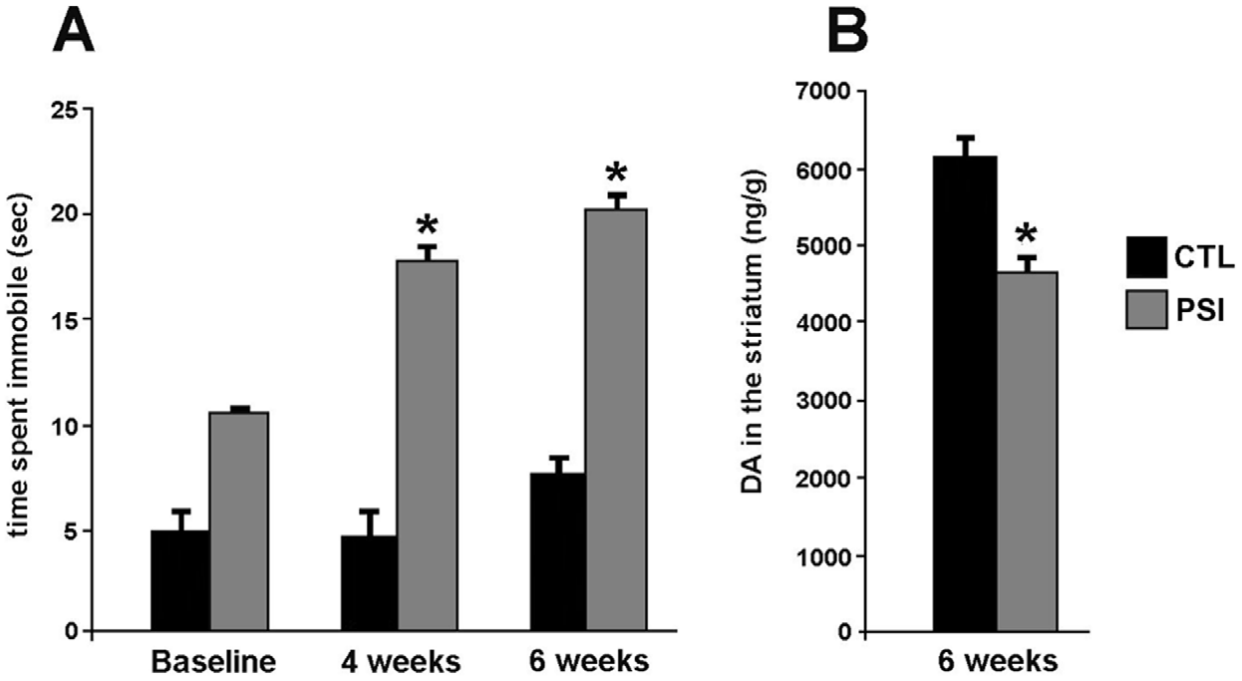
Motor performance assessment in treated animals. **Panel A** shows time (s) spent immobile at Tail suspension test in PSI-treated rats at 4 weeks (p = 0.03) and at 6 weeks (p = 0.02) as compared to baseline (grey bars). No change in motor performance was found in control animals (black bars). **Panel B** shows decreased DA levels (ng/g) in the nucleus striatum of PSI-treated rats at 6 weeks after initial injection (p = 0.02) (grey bars) as compared to controls (black bars).

Accompanying MR modifications, an impaired locomotor activity manifested as an increase in time spent immobile (s) over 5 minutes in the tail suspension test, evident at 4 week after the end of PSI treatment (p = 0.03) and more pronounced at 6 weeks after the end of PSI treatment (p = 0.02) as compared to baseline (**Figure 6** **, panel A**).

An impaired performance in treadmill test was apparent in PSI-treated rats, but did not reach significance.

No change in motor performance was observed in vehicle-treated rats as compared to baseline.

### Dopamine and Dopamine Metabolite Level HPCL Measurement

Decreased levels of DA in the striatum were found at 6 weeks after the end of PSI treatment as compared to controls (p = 0.02, **Figure 6** **, panel B**); a decrement was also observed in the level of the dopamine metabolite DOPAC in the PSI-treated rats, but did not reach statistical significance (p = 0.07). DOPAC/DA ratio was unchanged in the PSI-treated rats as compared to controls, highlighting that reduction of DA level in the striatum of PSI-treated rats was not attributable to increased DA metabolism.

### Immunofluorescence Analysis

**Figure 7:**
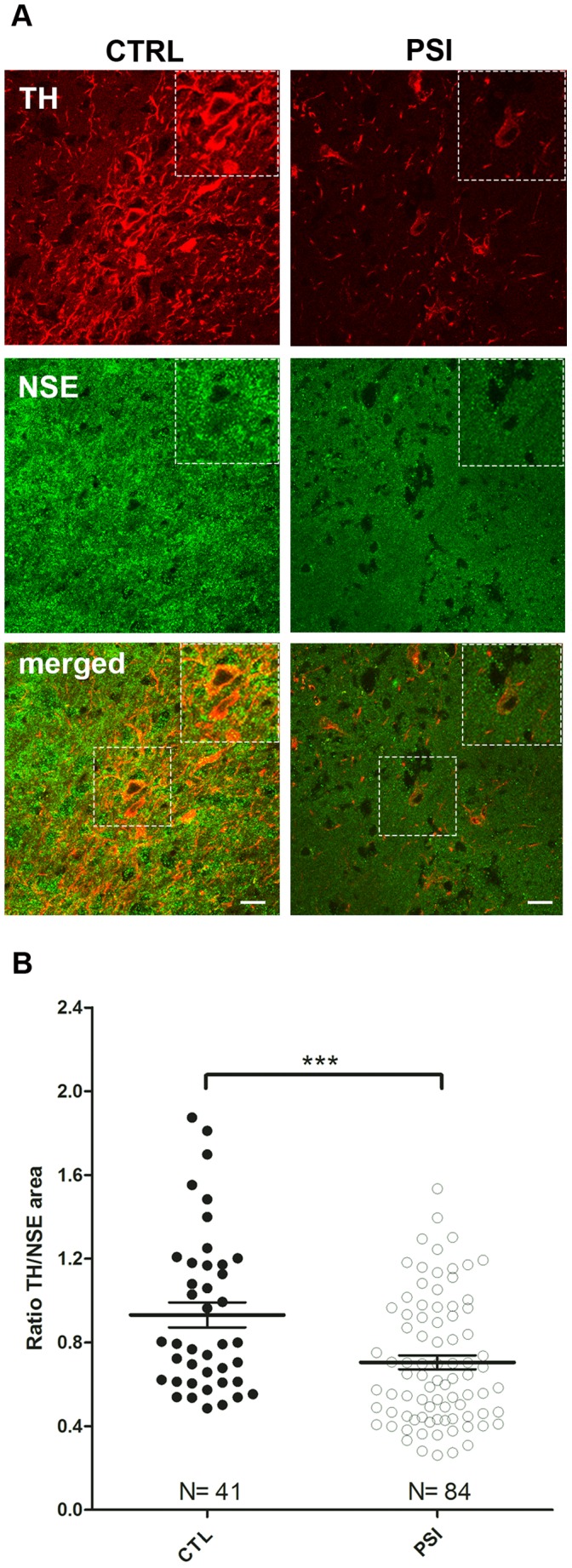
Immunofluorescence analysis. **Panel A:** Representative images of samples from control (CTL) and PSI (PSI) treated rats. Note the reduction of TH positive cells in PSI treated samples. **Panel B:** Data deriving from quantitative analysis of TH- and NSE-positive areas are expressed as ratio TH/NSE (p = 0.006).

Data deriving from quantitative analysis of TH- and NSE-positive areas (**supplementary** **figure 3**), expressed as ratio TH/NSE, showed a reduction of TH/NSE in PSI treated rats as compared to control rats (p = 0.006, **Figure 7**).

## Discussion

Controversial results of the different studies on the PSI-induced PD model seem not to have reduced the appeal of the concept of protein accumulation as an important pathophysiological hallmark of neurodegenerative disorders, including PD [24].

The interest in replicating the original findings by Mc Naught and colleagues [2] is still high as highlighted by recent reports, attempting to overcome possible technical problems claimed to be responsible for previous inconsistent results [12], [25]–[27].

In our study, we found that rats exposed subcutaneously to PSI developed by 4 weeks after treatment, significant difficulty with motor tasks progressively increasing overtime.

As in PD, these symptoms likely represent the downstream effect of a pathological cascade resulting in the degeneration of midbrain dopaminergic neurons of the SN pars compacta (SNpc) projecting to the nucleus striatum, the main input station of the basal ganglia neural circuit [28].

In keeping with these concepts, and according with recent MRI studies showing a significant degeneration of SN in PD patients [29]–[30], we found, at 4 weeks following PSI treatment, a significant size reduction of the SN, matched by immunocytochemistry findings, showing a loss of dopaminergic neurones in the SN.

Although MRI showed an apparently small (6%, nevertheless significant with p = 0.02) reduction of SN area at 4 weeks following PSI treatment, this tissue loss was demonstrated by immunofluorescence to specifically involve the SN dopaminergic neurons. Thus, the MRI result could be considered stronger than it might appear.

In addition, at 6 weeks after treatment, striatal dopamine levels had decreased significantly in the PSI-treated animals as compared to controls.

Some studies investigated *in vivo* by ^1^H-MRS the biochemical changes on striata. ^1^H-MRS allows to assess neuronal loss and neurodegeneration using substances such as NAA, Glx, tCho.

In our study, the morphometric change in the SN was accompanied by biochemical modifications at the striatum, suggesting that brain areas relevant in PD pathogenesis were affected by the PSI treatment.

In particular, in accordance with previous studies on humans [31] or on different animal models of PD [32], [33], we found a reduction of NAA/tCr. NAA is synthesized in the neuronal mitochondria and transported along axons and its concentration is reduced in case of neuronal loss [34], [35].

According with some PD studies on animal [36], tCr was stable. In this context, for comparability with numerous former MRS studies on PD [32]–[33] and to preserve a good signal to noise ratio (considering the use of clinical scanner and of a MRS voxel size <1 cm^3^), the ^1^H-MRS data were expressed as metabolite/tCr ratio by using water signal suppressed spectra.

The use of water signal suppressed spectra compared to the water signal unsuppressed spectra improve the assessment of the signal of some metabolites of interest such as Glx complex.

In a combined DTI and MRS study [40], patients with PD showed an increase of Glx/tCr ratio in lentiform nucleus and a reduction of fractional anisotropy in the rostral SN. These finding correlated with severity of motor impairment as measured by the Unified Parkinson Disease Rating Scale (UPDRS).

In our study, the ratio between GLX (mainly including glutamate and glutamine) [37] and tCr (Glx/tCr) was increased after PSI treatment. There are conflicting results about the role of Glx in PD [38], and while some ^1^H-MRS studies on PD showed no changes for Glutamate and Glutamine in the human striatum [39] and in rat models of PD [32], other authors showed by ^1^H-MRS high levels of Glx in the striatum of MPTP-intoxicated mice and hypothesized that such an increase, explainable as due to increased Glutamate-Glutamine cycling [36], might perform a protective action from Glutamate excitotoxic injury.

### Conclusions

The morphological and metabolic MR modifications after PSI treatment showed surprising similarities with findings in PD patients and invite to 1. reconsider the PSI-based model for further experimental assessments and to 2. evaluate MR techniques as surrogate markers for the study of the effects of PSI on the nigro-striatal pathway.

MRI and MRS techniques are particularly valuable to assess *in-vivo* dynamic changes in the nigro-striatal pathway overtime, in correlation with appearance of motor symptoms, giving possible useful information on disease progression (degree of SN volumetric changes, brain biochemical changes) and on mechanisms of response to pharmacological treatment, including efficacy and side effects.

## Supporting Information

Figure S1 **Intra- and inter- rater reliability tests.** Inter- rater reliability test was performed by asking two different experienced readers (reader 1 and 2) to perform the MR data analysis at baseline (time 1) and after treatments (with either PSI, grey bars or vehicle, black bars) (time 2). Intra-rater reliability was tested by asking each of the two different experienced readers to perform the MR data analysis after the first MR acquisition and to repeat it with a fifteen days delay. Results were analyzed respectively by Kruskal-Wallis non-parametric test followed by post hoc comparison using Wilcoxon and Mann-Whitney tests. The comparisons showed good reliability of our estimate.
(TIFF)

Figure S2 **^1^H-MRS total creatine (tCr) levels in the nucleus striatum of treated animals.** Box and Whiskers plots describe the distribution of the tCr values in the nucleus striatum quantified by using unsuppressed water signal as internal reference at baseline and at 4 weeks after treatment. Results from control animals are represented as white boxes (CTL, n = 5), results from PSI-treated animals are represented as grey boxes (PSI, n = 10). The bottom and top of the boxes show respectively the lower and upper quartiles; the bold band is the median; the ends of the whiskers show the minimum and the maximum value.
(TIF)

Figure S3 **Immunofluorescence analysis of NSE and TH-covered areas in the SN of treated animals.** Panel A shows NSE positive areas in PSI and vehicle-treated animals. Panel B shows TH- positive areas of PSI and vehicle-treated animals.
(TIF)
