# Supplementary figures and images for: Morphological and Metabolic Changes in the Nigro-Striatal Pathway of Synthetic Proteasome Inhibitor (PSI)-Treated Rats: A MRI and MRS Study
Source: PLoS One. 2013 Feb 19;8(2):e56501. doi: 10.1371/journal.pone.0056501 (PMC3576393; doi:10.1371/journal.pone.0056501)

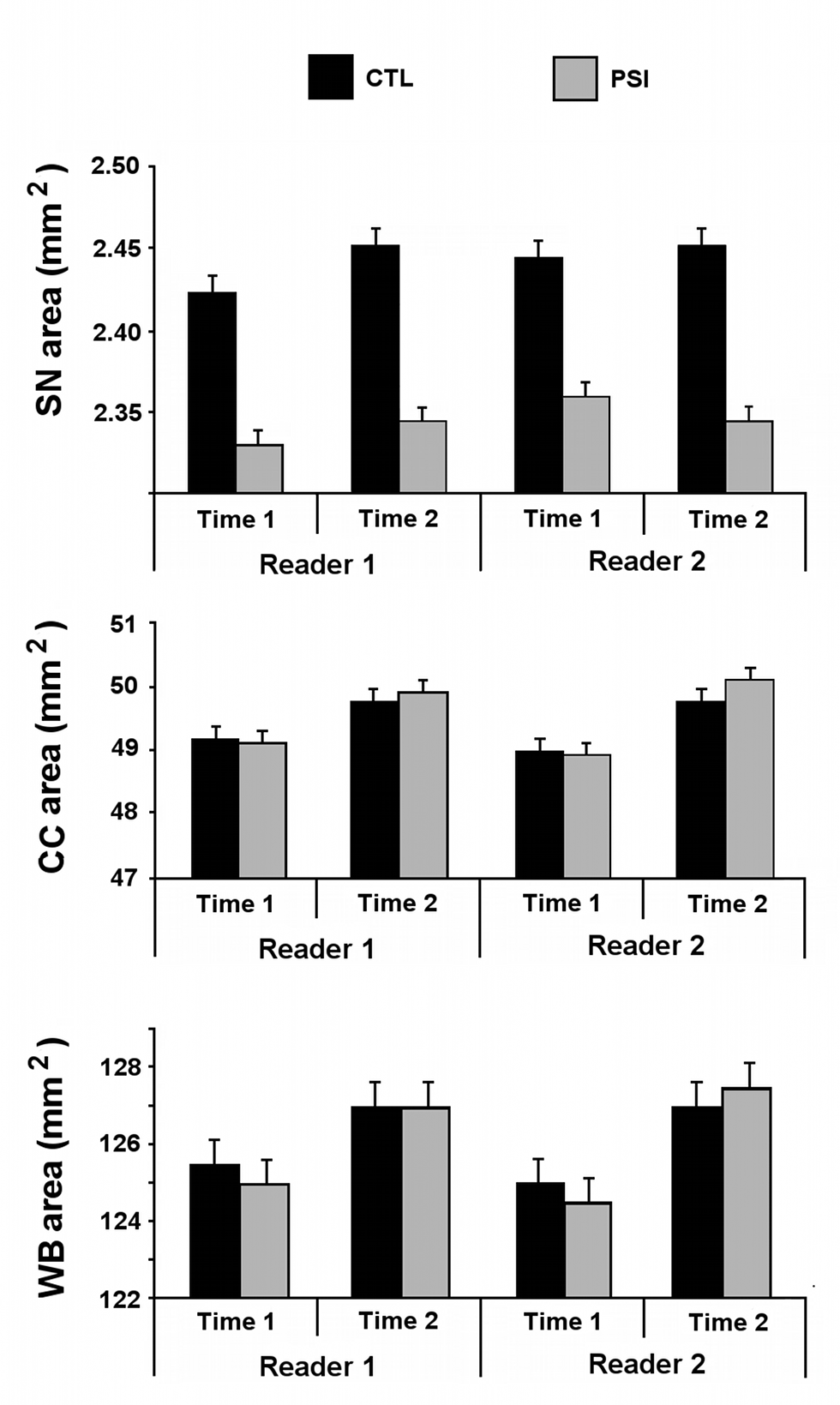

Supplement: Figure S1 — Intra- and inter- rater reliability tests. Inter- rater reliability test was performed by asking two different experienced readers (reader 1 and 2) to perform the MR data analysis at baseline (time 1) and after treatments (with either PSI, grey bars or vehicle, black bars) (time 2). Intra-rater reliability was tested by asking each of the two different experienced readers to perform the MR data analysis after the first MR acquisition and to repeat it with a fifteen days delay. Results were analyzed respectively by Kruskal-Wallis non-parametric test followed by post hoc comparison using Wilcoxon and Mann-Whitney tests. The comparisons showed good reliability of our estimate. (TIFF) [file pone.0056501.s001.tif]

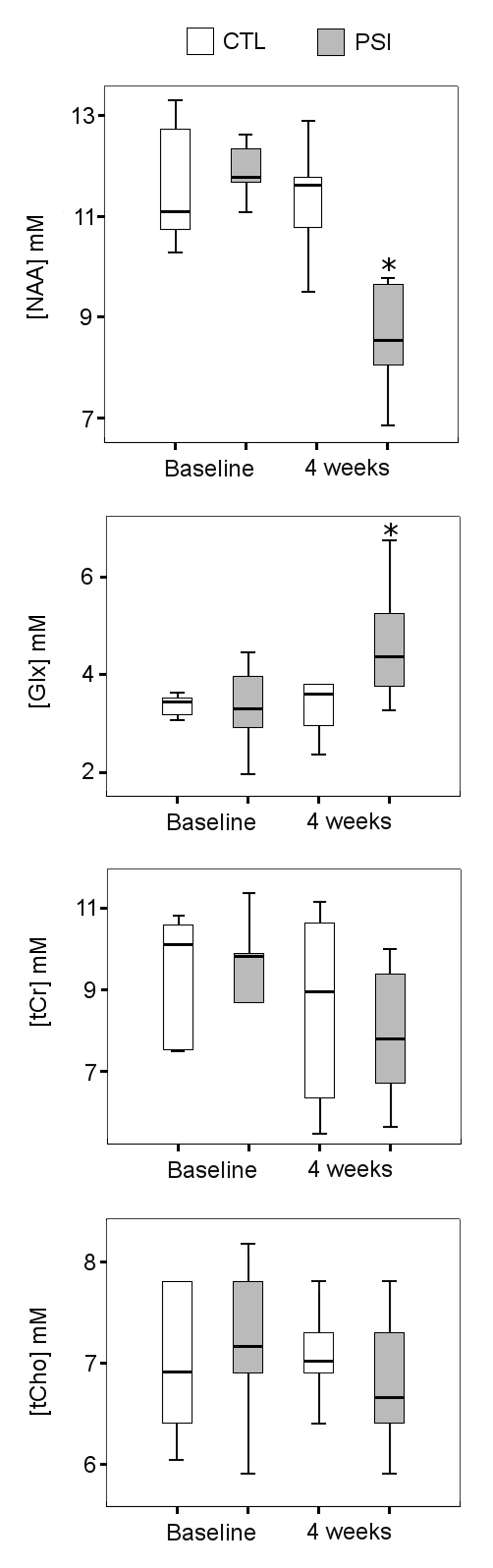

Supplement: Figure S2 — 1H-MRS total creatine (tCr) levels in the nucleus striatum of treated animals. Box and Whiskers plots describe the distribution of the tCr values in the nucleus striatum quantified by using unsuppressed water signal as internal reference at baseline and at 4 weeks after treatment. Results from control animals are represented as white boxes (CTL, n = 5), results from PSI-treated animals are represented as grey boxes (PSI, n = 10). The bottom and top of the boxes show respectively the lower and upper quartiles; the bold band is the median; the ends of the whiskers show the minimum and the maximum value. (TIF) [file pone.0056501.s002.tif]

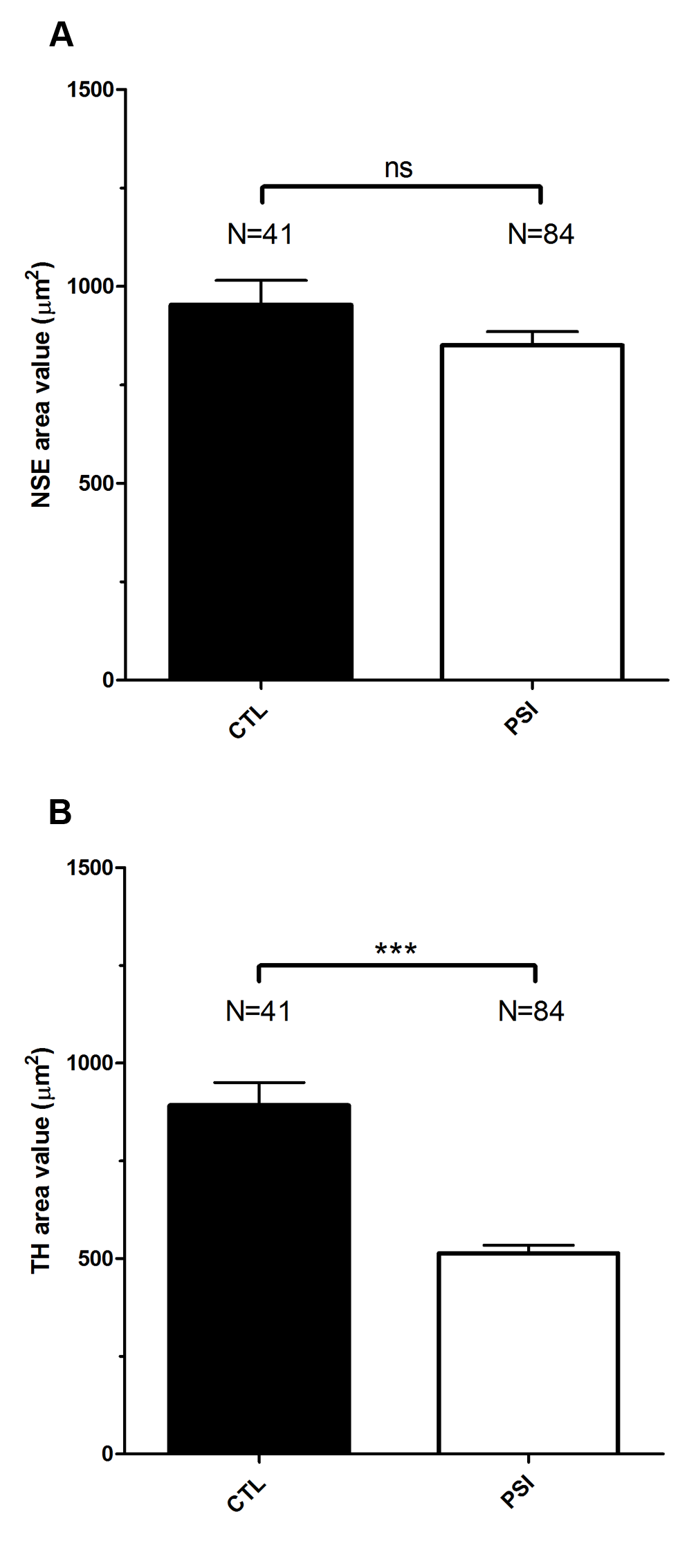

Supplement: Figure S3 — Immunofluorescence analysis of NSE and TH-covered areas in the SN of treated animals. Panel A shows NSE positive areas in PSI and vehicle-treated animals. Panel B shows TH- positive areas of PSI and vehicle-treated animals. (TIF) [file pone.0056501.s003.tif]
